# Novel localization of folate transport systems in the murine central nervous system

**DOI:** 10.1186/s12987-022-00391-3

**Published:** 2022-11-23

**Authors:** Vishal Sangha, Md. Tozammel Hoque, Jeffrey T. Henderson, Reina Bendayan

**Affiliations:** Leslie Dan Faculty of Pharmacy, University of Toronto, Toronto, Canada

**Keywords:** Folates, Reduced folate carrier, Proton-coupled folate transporter, Folate receptor, Blood–brain barrier, Blood–cerebrospinal fluid barrier, Arachnoid barrier, Cerebral folate deficiency

## Abstract

**Background:**

Folates are a family of B9 vitamins that serve as one-carbon donors critical to biosynthetic processes required for the development and function of the central nervous system (CNS) in mammals. Folate transport is mediated by three highly specific systems: (1) folate receptor alpha (FRα; *FOLR1*/*Folr1*), (2) the reduced folate-carrier (RFC; *SLC19A1/Slc19a1*) and (3) the proton-coupled folate transporter (PCFT; *SLC46A1/Slc46a1*). Folate transport into and out of the CNS occurs at the blood–cerebrospinal fluid barrier (BCSFB), mediated by FRα and PCFT. Impairment of folate transport at the BCSFB results in cerebral folate deficiency in infants characterized by severe neurological deficiencies and seizures. In contrast to the BCSFB, CNS folate transport at other brain barriers and brain parenchymal cells has not been extensively investigated. The aim of this study is to characterize folate transport systems in the murine CNS at several known barriers encompassing the BCSFB, arachnoid barrier (AB), blood–brain barrier (BBB) and parenchymal cells (astrocytes, microglia, neurons).

**Methods:**

Applying immunohistochemistry, localization of folate transport systems (RFC, PCFT, FRα) was examined at CNS barriers and parenchymal sites in wildtype (C57BL6/N) mice. Subcellular localization of the folate transport systems was further assessed in an in vitro model of the mouse AB. Gene and protein expression was analyzed in several in vitro models of brain barriers and parenchyma by qPCR and western blot analysis.

**Results:**

RFC, PCFT, and FRα expression was localized within the BCSFB and BBB consistent with previous reports. Only RFC and PCFT expression was detected at the AB. Varied levels of RFC and PCFT expression were detected in neuronal and glial cells.

**Conclusions:**

Localization of RFC and PCFT within the AB, described here for the first time, suggest that AB may contribute to folate transport between the peripheral circulation and the CSF. RFC and PCFT expression observed in astrocytes and microglia is consistent with the role that one or both of these transporters may play in delivering folates into cells within brain parenchyma. These studies provide insights into mechanisms of folate transport in the CNS and may enhance our understanding of the critical role folates play in neurodevelopment and in the development of novel treatment strategies for disorders of brain folate deficiency due to impaired transporter function.

**Supplementary Information:**

The online version contains supplementary material available at 10.1186/s12987-022-00391-3.

## Introduction

There are complex transport processes within unique barriers in the central nervous system (CNS) that regulate the availability of a variety of micronutrients required for neural development and function. These include the blood–brain barrier (BBB), the blood–cerebrospinal fluid barrier (BCSFB) at the choroid plexus (CP), and the less studied arachnoid barrier (AB) (or blood–arachnoid barrier) [1, 36]. These barriers limit paracellular transport of molecules into the CNS at tight junctions populated with claudins, occludins (and others) , and at endothelial/epithelial and ependymal cell interfaces. There are a variety of transporters belonging to the ATP-binding cassette (ABC), solute carrier (SLC) superfamilies and channels expressed at these sites providing highly specialized control of nutrients, metabolites and fluid that enter and exit the CNS [1, 11, 25, 36]. Transporters within cells of the brain parenchyma (i.e., astrocytes, microglia, neurons) provide further highly specific mechanisms by which substances are delivered to neural tissues [7, 36]. Together, these cells within physical and biochemical barriers work in concert to regulate CNS homeostasis.

Folates represent a family of B9 vitamins that serve as one-carbon donors required for DNA, RNA, and amino acid synthesis and for the regulation of methylation reactions in the CNS and tissues in general [13, 24]. Folates are bivalent anions at physiological pH and highly hydrophilic so that their passive diffusion is limited, requiring the need for specific mechanisms to achieve transport across cell membranes [42]. Transport of folates and folate analogs is mediated by three distinct mechanisms that vary in their affinity and optimal activity conditions. Folate receptor alpha (FRα) (gene: *FOLR1/Folr1*) is a high affinity glycosylphosphatidylinositol-membrane-anchored glycoprotein that mediates folate transport via receptor-mediated endocytosis, with optimal activity at neutral pH [22, 53]. The reduced folate carrier (RFC) (gene: *SLC19A1/Slc19a1*) functions as an antiporter that exchanges folates with cellular organic phosphates, with optimal activity at neutral pH. It is the major route of delivery of folates to systemic tissues [52, 54]. Finally, the proton-coupled folate transporter (PCFT) (gene: *SLC46A1/Slc46a1*) is a proton cotransporter with optimal activity at pH 5.5–6.0. PCFT is required for intestinal folate absorption and transport of folates across the BCSFB [31, 52].

Folate transport into the CSF occurs primarily at the BCSFB and is mediated by both FRα which is localized on the apical, and to a lesser extent the basolateral membranes of CP epithelial cells and PCFT which is located at the basolateral membrane [17, 52, 55]. The role of these transporters has been established by the cerebral folate deficiency (CFD) syndromes characterized by very low CSF folate levels that occur when there is loss of function mutations in PCFT [hereditary folate malabsorption (HFM)] or FRα [16, 23, 30]. RFC is also expressed at the apical membrane but its contribution to CNS folate homeostasis is unclear [17, 43]. CFD has also been associated with a variety of other disorders [30]. Autoantibodies against FRα (FRΑAs) appear to interfere with FRα function and are associated with autism spectrum disorder and other disorders such as Rett syndrome, Alpers’s syndrome, and Kearns–Sayre syndrome [14, 15, 19, 33, 34, 39].

Our laboratory has focused on the development of novel strategies for the treatment of disorders associated with impaired folate transport at the BCSFB. Our studies suggest that the BBB may be a useful alternative route for the delivery of folates to the brain when there is a loss of FRα or PCFT function [2–5]. We demonstrated that activation of the Vitamin D nuclear receptor (VDR) by its specific natural ligand 1,25-dihydroxyvitamin D3 (or calcitriol) resulted in increased brain folate delivery in vivo in mice lacking FRα [2]. More recently, we have shown that upregulation of nuclear respiratory factor 1 (NRF-1) by the pyrroloquinoline quinone (PQQ) ligand in hCMEC/D3 cells, an immortalized human brain microvascular cell line, increased expression and function of RFC [4]. These studies suggest that enhancing RFC-mediated folate transport at the BBB may be a promising therapeutic approach in disorders where folate transport at the CP is compromised. It is unclear as to whether AB may also contribute to brain folate uptake. Single-cell mouse RNAseq data indicates expression of RFC and PCFT in leptomeningeal cells of the AB consistent with the expression of a variety of other transporters at this site [20, 49, 50]. Likewise, it is unclear as to which transporters play an important role in folate delivery to brain parenchymal cells (i.e., astrocytes, microglia, neurons), which are the critical destination for CNS folate delivery. In this study we further characterize the localization of the folate transport systems (i.e., RFC, PCFT, FRα) in the various compartments and cells of the murine brain to provide further clues into the routes of folate transport that may contribute to overall brain folate sufficiency and may serve as alternative pathways for folate delivery when PCFT or FRα are deficient.

## Methods

### Materials

All reagents used for cell culture were of highest quality and purchased from Invitrogen (Carlsbad, CA, USA), unless indicated otherwise. For immunohistochemistry and immunocytochemistry analysis, primary rabbit polyclonal AE930 anti-RFC (1:50) was kindly provided by Dr. I.D. Goldman (Albert Einstein College of Medicine, NY, USA). Primary rabbit polyclonal anti-PCFT (ab25134), primary rabbit polyclonal anti-FRα (ab67422), primary mouse monoclonal anti-E-cadherin (ab231303), primary mouse monoclonal anti-GFAP (ab4648), and primary mouse monoclonal anti-NeuN (ab104224) antibodies were purchased from Abcam Biotechnology (Cambridge, MA, USA). Primary rat monoclonal anti-CD31 antibody (AF3628) was purchased from R&D Systems (Minneapolis, MN, USA). Primary mouse monoclonal anti-Aquaporin-1 (Aqp1) antibody (AB2219) was purchased from Sigma Aldrich (St. Louis, MO, USA). Primary mouse monoclonal anti-IBA1 antibody (019-19741) was purchased from FUJIFILM Wako Chemicals (Richmond, VA, USA). Primary mouse monoclonal anti-Vimentin (sc-6260) and primary mouse monoclonal anti-Na+/K+-ATPase α (sc-58628) were obtained from Santa Cruz Biotechnology (Dallas, TX, USA). Real-time quantitative polymerase chain reaction (qPCR) reagents, including reverse transcription cDNA kits were ordered from Applied Biosystems (Foster City, CA, USA). Primers for qPCR analysis were purchased from Life Technologies (Carlsbad, CA, USA). For western blot analysis, primary rabbit polyclonal anti-SLC19A1 (AV44167), and primary rabbit polyclonal anti-SLC46A1 (PCFT) antibody (SAB2108339) were obtained from Sigma Aldrich. Primary rabbit monoclonal anti-FRα antibody (ab221543) was purchased from Abcam Biotechnology. Mouse monoclonal anti-β-actin antibody (sc-517582) was purchased from Santa Cruz Biotechnology (Dallas, TX, USA). Anti-rabbit Alexa Fluor 594 and anti-mouse Alexa Fluor 488–conjugated secondary antibodies were purchased from Invitrogen.

### Cell culture

Primary cultures of mouse brain microvascular endothelial cells (C57BL/6N strain) were kindly obtained from Dr. Isabel Aubert, Sunnybrook Health Science Center, Toronto, ON, and prepared as previously described by Alam et al. [2]. Briefly, cells were grown in 75 cm^2^ gelatin-coated flasks and were cultured in mouse endothelial cell basal medium supplemented with vascular endothelial growth factor, endothelial cell growth supplement, heparin, epidermal growth factor, hydrocortisone, L-glutamine, fetal bovine serum (FBS), and antibiotic–antimycotic solution at 37 °C in 5% CO_2_/95% air (Cell Biologics, Chicago, IL, USA). Upon reaching confluence, cells were collected and further processed for qPCR and western blot analysis [2]. Immortalized mouse AB cells were kindly provided by Dr. Erin Schuetz (St. Jude’s Children’s Research Hospital, Memphis, TN, USA) and were cultured in 75 cm^2^ poly-d-lysine (PDL) coated flasks in DMEM supplemented with 9% FBS, 1% horse serum, 1× penicillin/streptomycin, 1× fungizone, and 1× glutamine as previously described [49]. Upon reaching confluence, cells were collected for qPCR and western blot analysis. AB cells were characterized by analyzing gene expression of the standard epithelial markers desmoplakin and vimentin, as well as examining vimentin immunofluorescence (Additional file 1: Fig. S1). Primary cultures of CD1 mouse hippocampal/cortical neurons were kindly provided by Dr. Beverly Orser (Department of Anesthesiology and Pain Medicine, University of Toronto, Canada) and were prepared as previously described [8]. In brief, neurons were obtained from the hippocampus and cortex of fetal mice pups [embryonic day (ED) 18] which were later processed and plated on 35-mm culture dishes coated with collagen. Neurons were grown in a neurobasal medium supplemented with 2% B27 and 1% Glutamax [8]. Upon reaching confluence, cells were collected for qPCR and western blot analysis. Primary cultures of neurons were characterized by analyzing gene expression of the standard neuronal marker NeuN [44]. Primary cultures of mouse mixed astrocytes and microglia (or mixed glial cells) were prepared in our laboratory as previously described [27]*.* In brief, cerebral cortices were isolated from 1–2-day old C57BL6/N mice pups, and further processed to generate a mixed glial suspension. Mixed glial cells from four brains were plated onto 75 cm^2^ PDL-coated tissue flasks and incubated in DMEM (Wisent Inc, Montreal, QC, Canada) supplemented with 10% heat inactivated FBS, and 1× penicillin–streptomycin at 37 °C in 5% CO_2_/95% air. Upon reaching confluence, cells were collected and further processed for qPCR and western blot analysis.

### Isolation of mouse CP tissue

CP was isolated from adult C57BL6/N mice (8–12 weeks old). Animals were decapitated, and brains were immediately removed and placed at room temperature in a high glucose Dulbecco’s modified Eagle’s medium (DMEM) for dissection (Thermo Fisher Scientific, Waltham, MA, USA). Intact CP tissue was removed from the lateral and fourth ventricle. Due to limited sample volume, tissues obtained from three animals were pooled for real-time quantitative PCR (qPCR) and western blot analysis. To confirm the presence of CP epithelial cells, gene expression of the CP epithelial marker aquaporin-1 (Aqp-1) was analyzed [28].

### Immunohistochemistry analysis

Localization of the folate transport systems was assessed in frozen brain sections of C57BL6/N mice. Mice (8–12 weeks of age) were administered 2–5% isoflurane to induce anesthesia. Animals were initially perfused with 30 mL of phosphate buffer saline (PBS) solution and then perfused with 30 mL of 4% paraformaldehyde (PFA) through the posterior end of the left ventricle. Whole brains were dissected immediately following perfusion and were fixed in 4% PFA overnight at 4 °C. Brains were then rinsed with PBS and transferred to a gradient of 10%, 20%, and 30% sucrose. Following the sucrose gradient, brains were placed in cryomolds filled with optimal cutting temperature compound and submerged in liquid nitrogen until frozen. Coronal cryostat sections 20-microns thick were prepared from whole brains and transferred to microscope slides. Primary rabbit polyclonal AE930 anti-RFC (1:50), anti-PCFT (1:50) (ab25134), and anti-FRα (1:50) (ab67422) antibodies were used to detect RFC, PCFT, and FRα respectively. The following cell markers were used to examine folate transporter/receptor localization: rat monoclonal anti-CD31 (BBB) (1:50) (AF3628), mouse monoclonal anti-E-cadherin (AB) (1:50) (ab231303), mouse monoclonal anti-Aquaporin-1 (Aqp-1) (CP) (AB2219) (1:100), mouse monoclonal anti-GFAP (astrocytes), mouse monoclonal anti-NeuN (neurons) (1:100) (ab104224), and (1:50) (ab4648), and mouse monoclonal anti-IBA1 (microglia) (1:100) (019-19741). Following primary antibody incubation, slides were incubated with anti-rabbit Alexa Flour 594 or anti-rabbit Alexa Flour 488, and anti-rat Alexa Flour 594 or anti-mouse Alexa Flour 488-conjugated secondary antibodies (1:400). As a negative control, slices were incubated with only secondary antibody to confirm specificity of primary antibody staining. Slices were visualized using an LSM 700 laser-scanning confocal microscope (Carl Zeiss AG) at a 63× objective lens operated with ZEN Software.

### Immunocytochemistry analysis

Subcellular localization of the folate transport systems was examined in immortalized mouse AB cells. Cells were grown as a monolayer in PDL-coated glass coverslips and fixed with 4% PFA for 20 min, followed by permeabilization with 0.1% Triton X-100. Non-specific sites were blocked with 0.1% [mass/volume (m/v)] bovine serum albumin (BSA) and 0.1% m/v skim milk solution. Primary rabbit polyclonal AE930 anti-RFC (1:50) and anti-PCFT (1:50) (ab25134) antibodies were used to detect RFC and PCFT, respectively. Mouse monoclonal anti-Na^+^/K^+^-ATPase α (sc-58628) (1:50) was used to visualize the plasma membrane, with mouse monoclonal vimentin (1:50) used an additional cell surface marker [49]. Following primary antibody incubation, cells were incubated with anti-rabbit Alexa Flour or anti-mouse Alexa Flour 488-conjugated secondary antibodies (1:500). As a negative control, cells were incubated with only secondary antibody to confirm specificity of primary antibody staining. Cells were visualized using an LSM 700 laser-scanning confocal microscope (Carl Zeiss AG) at a 63× objective lens operated with ZEN Software.

### Gene expression analysis

mRNA expression of the various genes of interest was assessed in cells and tissue using qPCR analysis. Total RNA was isolated from mouse CP, primary cultures of mouse brain microvascular endothelial cells, immortalized AB cell cultures, primary cultures of mouse neurons, and primary cultures of mouse mixed glial cells using TRIzol and treated with DNase I to remove any contaminating genomic DNA. RNA concentration (absorbance at 260 nm) and purity (absorbance ratio 260/280) was assessed using NanoDrop One Spectrophotometer (Thermo Fisher Scientific). Following isolation, RNA (2 μg) was reverse transcribed to cDNA using a high-capacity reverse transcription cDNA kit according to manufacturer’s instructions. Specific mouse primers for: *Slc19a1* (RFC; Mm00446220_m1), *Slc46a1* (PCFT; Mm00546630_m1), *Folr1* (FRα; Mm00433355_m1), *Gfap* (GFAP; Mm01253033_m1), *Aif1* (Iba1; Mm00479862_g1), *Dsp* (Desmoplakin; Mm01351876_m1); *Vim* (Vimentin; Mm01333430_m1); *Rbfox3* (NeuN; Mm01248781_m1); *Aqp1* (Aquaporin-1; Mm01326466_m1) were obtained from Life Technologies for use with TaqMan qPCR chemistry. All gene expression assays were performed in triplicates using the housekeeping gene cyclophilin B as the internal control. For each gene of interest, relative mRNA expression was determined by calculating the difference in CT values (ΔCT) between the target gene and cyclophilin B.

### Western blotting

Western blot analysis was performed according to our previously published protocol [3]. Briefly, tissue or cell lysates were obtained after lysing samples with a modified RIPA buffer containing the following: 50 mM of Tris pH 7.5, 150 mM of NaCl, 1 mM of EGTA, 1 mM of sodium o-vanadate, 0.25% (v/v) of sodium deoxycholic acid, 0.1% (v/v) of sodium dodecyl sulfate (SDS), 1% (v/v) of NP-40, 200 μM of PMSF, and 0.1% (v/v) of protease inhibitor. Total protein concentrations of cell and tissue lysates were quantified using Bradford’s protein assay (Bio-rad Laboratories, Hercules, CA, USA) with BSA as a standard. For each sample, total protein (15 or 50 μg) was mixed with 1× Laemmli buffer and 10% β-mercaptoethanol and separated on 10% SDS–polyacrylamide gel, and later electro-transferred onto a polyvinylidene fluoride membrane overnight at 4 °C. To reduce non-specific binding, blots were incubated with 5% skim milk prepared in tris-buffered saline solution containing 0.1% Tween 20 for 1 h at room temperature. Blots were then incubated overnight at 4 °C with primary rabbit polyclonal anti-SLC19A1 (RFC) antibody (1:250, AV441671848995), rabbit polyclonal anti-SLC46A1 (PCFT) antibody (1:250, SAB2108339), rabbit monoclonal anti-FRα antibody (1:500, ab221543) or mouse monoclonal anti-β-actin antibody (1:1000, sc-517582). Blots were incubated for 1.5 h with corresponding horseradish peroxidase-conjugated anti-rabbit (1:5000) or anti-mouse (1:5000) secondary antibody. The protein bands were detected using enhanced chemiluminescence SuperSignal West Pico System (Thermo Fisher Scientific) and imaged using the ChemiDoc MP Imaging System (Bio-Rad).

## Results

### Localization of the folate transport systems in the mouse CNS

**Fig. 1:**
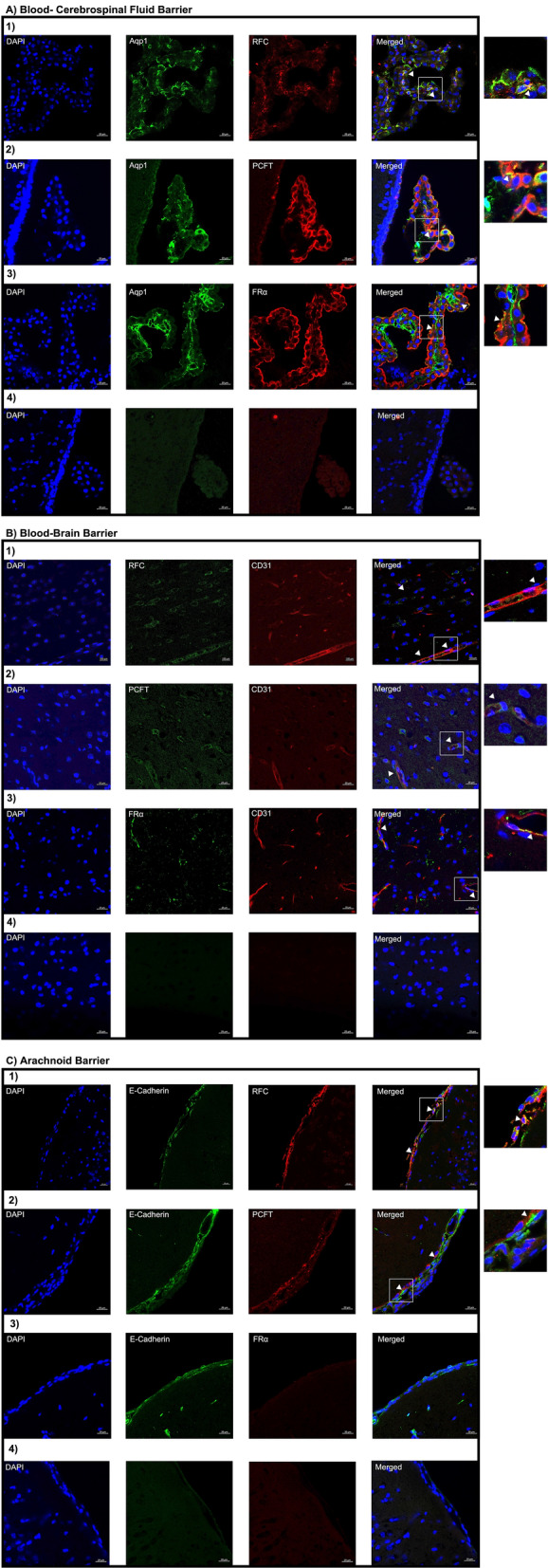
Localization of the folate transport systems at the brain barriers: **A** epithelial cells of the BCSFB, **B** microvessel endothelial cells of the BBB, **C** epithelial cells of the AB. Mouse brain sections were stained with the following: DAPI nuclear marker, AE390 anti-RFC (1:50) (Panel 1), anti-PCFT (1:50) (Panel 2), or anti-FRα (1:50) (Panel 3). The following cell markers were used to determine cell-specific localization: anti-Aqp-1 (1:100) (CP epithelial marker), anti-CD31 (1:50) (BBB endothelial marker), or anti-E-Cadherin (1:50) (AB epithelial marker). No primary antibody was used as a negative control (Panel 4). Arrows denote localization of RFC, PCFT, or FRα with cell markers. Sections were visualized using confocal microscopy (LSM 700; Carl Zeiss) operated with ZEN software using an oil-immersion 63× lens

**Fig. 2:**
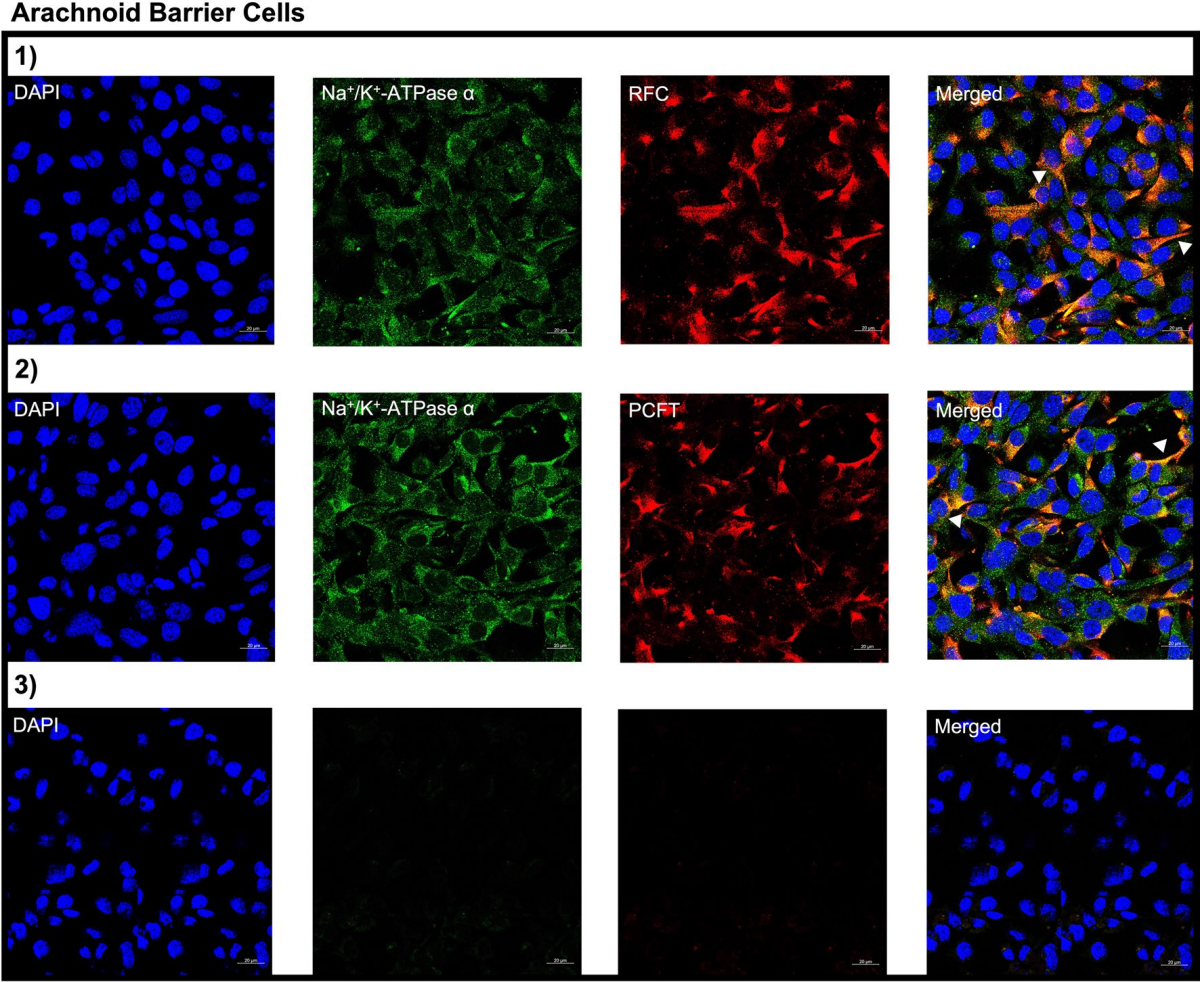
Cellular localization of RFC and PCFT in immortalized cell cultures of mouse AB. Cells were stained with the following: DAPI nuclear marker, AE390 anti-RFC (1:50) (Panel 1) or anti-PCFT (1:50) (Panel 2). To visualize the plasma membrane, cells were stained with the membrane marker Na^+^/K^+^-ATPase α (1:50). No primary antibody was used as a negative control (Panel 3). Arrows denote localization of RFC or PCFT with Na^+^/K^+^-ATPase α. Cells were visualized using confocal microscopy (LSM 700; Carl Zeiss) operated with ZEN software using an oil-immersion 63× lens

**Fig. 3:**
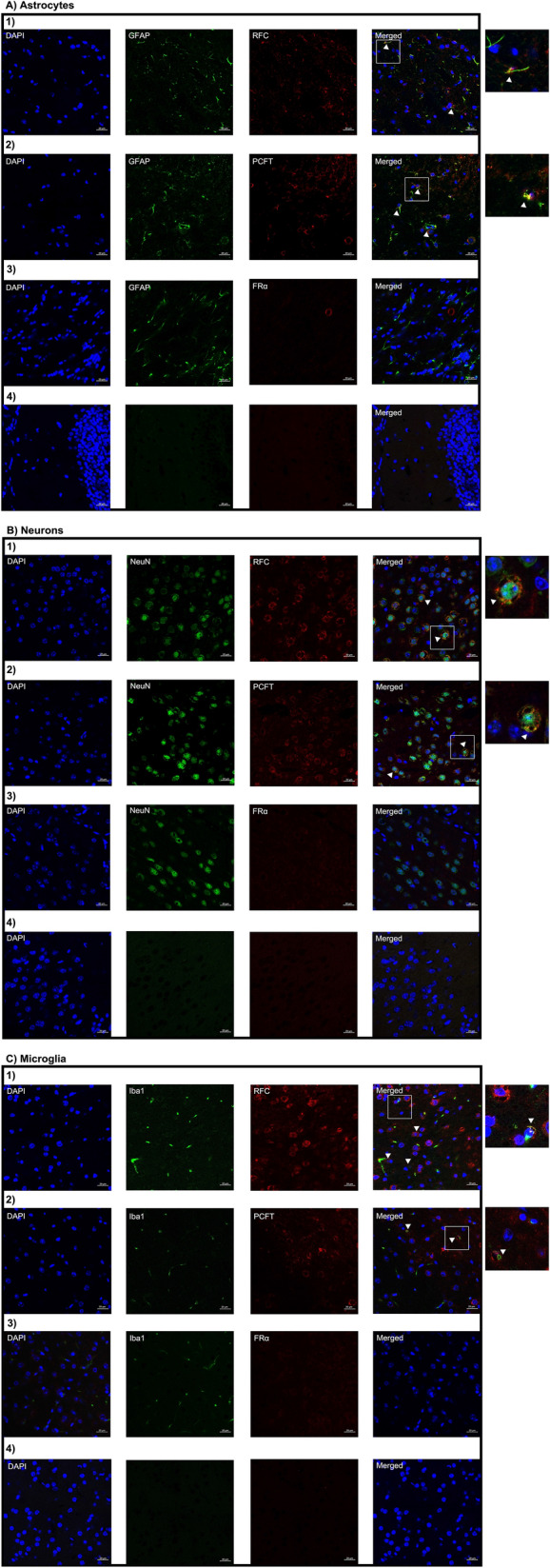
Localization of the folate transport systems in brain parenchyma: **A** astrocytes, **B** neurons, **C** microglia. Mouse brain sections were stained with the following: DAPI nuclear marker, AE390 anti-RFC (1:50) (Panel 1), anti-PCFT (1:50) (Panel 2), or anti-FRα (1:50) (Panel 3). The following cell markers were used to determine cell-specific localization: anti-GFAP (1:50) (astrocyte marker), anti-NeuN (1:100) (neuronal marker), or anti-IBA1 (1:100) (microglial marker). No primary antibody was used as a negative control (Panel 4). Arrows denote localization of RFC, PCFT, or FRα with cell markers. Sections were visualized using confocal microscopy (LSM 700; Carl Zeiss) operated with ZEN software using an oil-immersion 63× lens

Using immunohistochemistry we examined the localization of RFC, PCFT and FRα in the following brain barriers of the mouse CNS: BCSFB, BBB, and AB. Wildtype mice brain tissue sections were immuno-stained with antibodies against the folate transporters along with cell-specific markers and visualized using confocal microscopy. Immunohistochemical analysis revealed localization of RFC, PCFT, and FRα at the CP epithelium, stained with the CP epithelial membrane marker Aqp-1. Minor RFC localization is observed on the apical side of the CP epithelium, with PCFT localized on the basolateral membrane, and to a lesser extent in intracellular compartments (Fig. 1A). Lastly, FRα localization is primarily observed at the apical membrane, with minimal staining observed at the basolateral membrane (Fig. 1A). At the mouse BBB, mouse brain microvessel endothelial cells were stained with CD31, with localization of all three folate transport systems observed (Fig. 1B). At the AB, localization of RFC and PCFT (but not FRα) was detected in AB epithelial cells, which were stained with the standard epithelial marker e-cadherin (Fig. 1C). In addition, the imaging data reveals more robust RFC staining relative to PCFT at the AB (Fig. 1C). To confirm the membrane localization of RFC and PCFT in AB cells, immortalized AB cell cultures were immuno-stained with antibodies against RFC and PCFT, with anti-Na^+^/K^+^-ATPase α used as a plasma membrane marker. In AB cells, RFC and PCFT staining is detected, with similar localization to Na^+^/K^+^-ATPase α observed for both transporters (Fig. 2). The localization of RFC, PCFT and FRα was also investigated in brain parenchyma of wildtype mice using the following cell-specific markers: GFAP (astrocytes), NeuN (neurons), IBA1 (microglia). RFC and PCFT, but not FRα localization was detected in astrocytes, neurons, and microglia (Fig. 3). Stronger RFC and PCFT staining was observed in neurons (Fig. 3B) compared to astrocytes and microglia (Fig. 3A, C). Furthermore, the imaging data suggests that RFC and PCFT may exhibit membrane localization in neurons (Fig. 3B).

### Expression of the folate transport systems in the mouse CNS

**Fig. 4:**
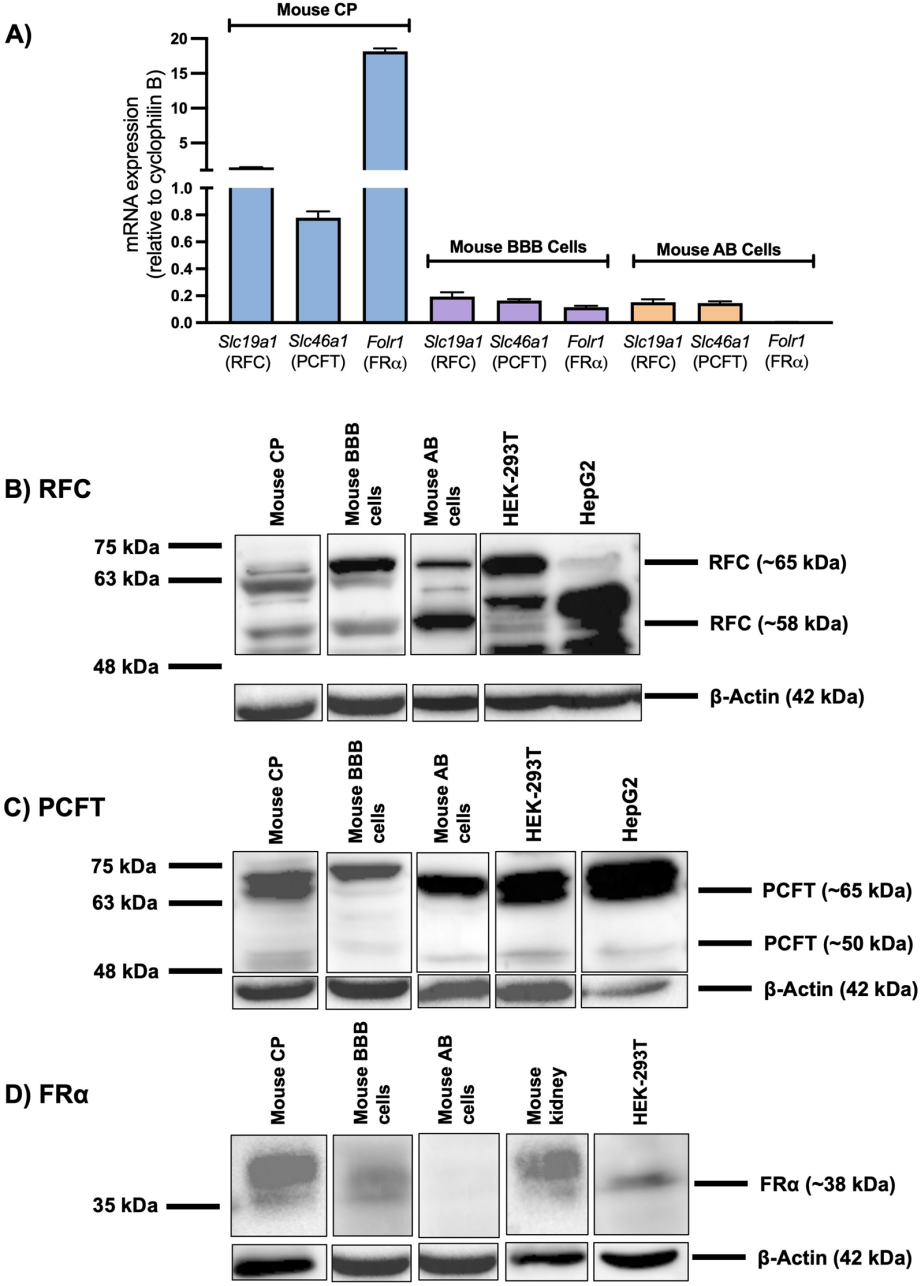
Relative expression of the folate transport systems in various models of the brain barriers. **A** Relative mRNA expression of mouse *Slc19a1* (RFC), *Slc46a1* (PCFT), and *Folr1* (FRα) was assessed in isolated mouse CP tissue (Mouse CP), primary cultures of mouse brain microvascular endothelial cells (Mouse BBB) and in immortalized mouse AB cells (Mouse AB). Results are presented as mean relative mRNA expression normalized to the housekeeping gene mouse cyclophilin B (n = 4). **B**–**D** Representative immunoblots from three separate experiments demonstrating protein expression of RFC, PCFT and FRα in isolated mouse CP tissue, in primary mouse brain microvascular endothelial cells, and in immortalized mouse AB cells. HEK293 and HepG2 cells were used as positive controls for the three transport systems, with mouse kidney used as an additional positive control for FRα. Multiple protein bands for RFC and PCFT represent differential glycosylation

**Fig. 5:**
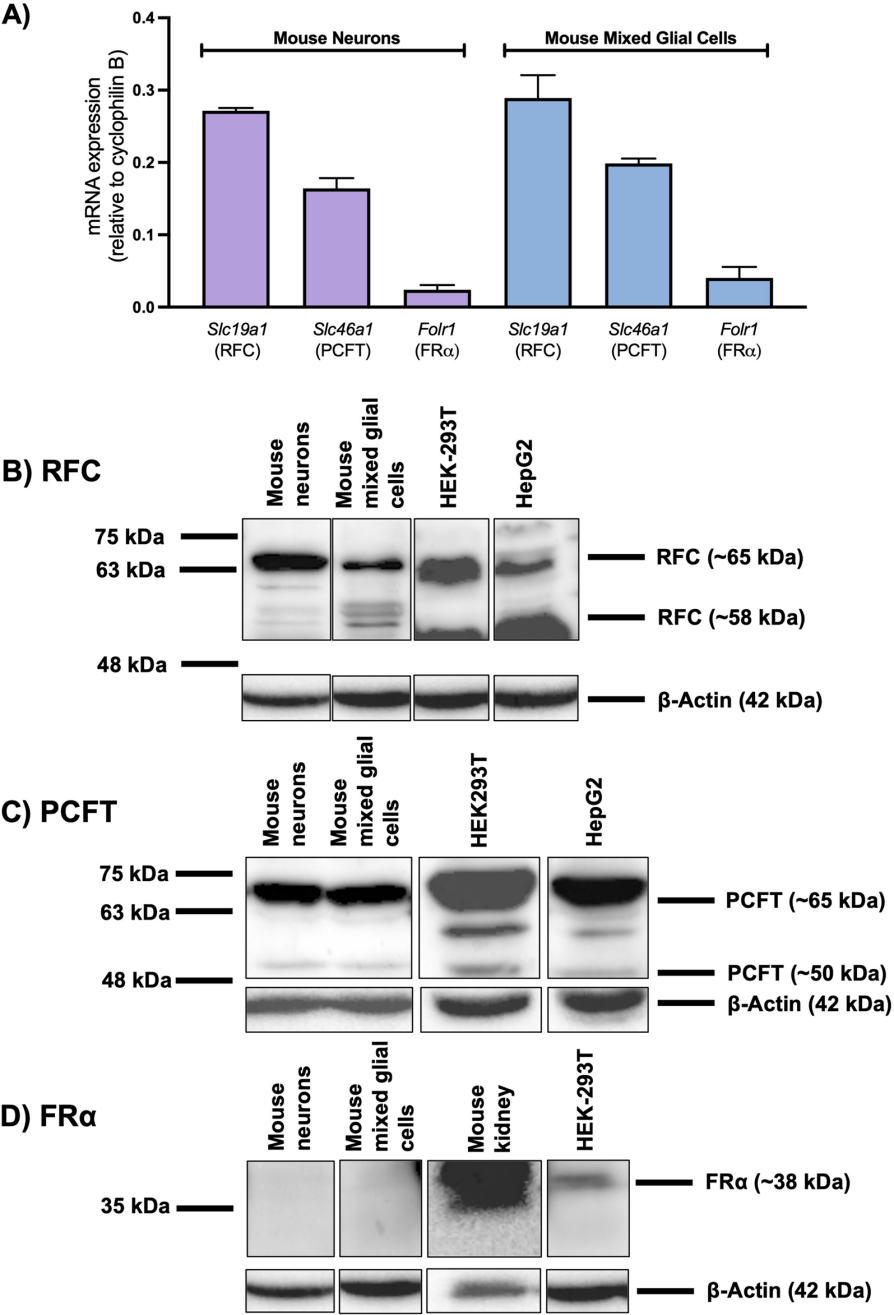
Relative expression of the folate transport systems in brain parenchymal cells. **A** Relative mRNA expression of mouse *Slc19a1* (RFC), *Slc46a1* (PCFT), and *Folr1* (FRα) was assessed in primary cultures of mouse neurons, and primary cultures of mouse mixed glial cells. Results are presented as mean relative mRNA expression normalized to the housekeeping gene mouse cyclophilin B (n = 4). **B**–**D** Representative immunoblots from three separate experiments demonstrating protein expression of RFC, PCFT, and FRα in primary cultures of mouse neurons, and in primary cultures of mixed glial cells. HEK293 and HepG2 cells were used as positive controls for the three transport systems, with mouse kidney used as an additional positive control for FRα. Multiple protein bands for RFC and PCFT represent differential glycosylation

In our in vitro models of brain barriers and brain parenchyma, relative gene and protein expression of the folate transport systems was documented through qPCR and western blot analysis, respectively. RFC and PCFT gene expression was detected in isolated mouse CP, primary cultures of mouse brain microvascular endothelial cells and in AB epithelial cells (Fig. 4A). However, FRα gene expression was only detected in mouse CP and in mouse brain microvascular endothelial cells (Fig. 4A). Corresponding western blots revealed protein expression of RFC and PCFT in the mouse CP, primary mouse brain microvascular endothelial cells, and AB epithelial cells, which is in agreement with the localization and gene expression data (Fig. 4B, C). FRα protein expression was detected in mouse CP and in mouse brain microvascular endothelial cells, which is consistent with the localization data demonstrating expression of FRα at the mouse BCSFB and BBB (Fig. 4D). In primary cultures of mouse neurons and in primary cultures of mouse mixed glial cells, gene expression of RFC and PCFT was detected, however low FRα gene expression was also observed (Fig. 5A). Western blot analysis revealed RFC and PCFT (but not FRα) protein expression in mouse neurons and in mixed glial cells, consistent with the localization and gene expression data (Fig. 5B–D).

## Discussion

Folates play a significant role in several biosynthetic processes and are critical in normal CNS growth and development [5]. The BCSFB has been uncovered as the primary route of folate uptake in the brain, through the concerted action of FRα and PCFT [17, 52, 55]. FRα has been proposed to function as the primary transport system responsible for the transcytosis of folates across the CP epithelium, with localization observed primarily on the apical membrane, and to a lesser extent on the basolateral side [17, 29, 38]. Despite this localization pattern, evidence of blood-to-CSF transport of folates has been observed at the CP epithelium, with the study by Grapp et al., demonstrating FRα-mediated basolateral to apical transport of labelled folates in rat CP Z310 cells [17]. In addition, Wollack et al., highlighted the role of FRα in mediating folate uptake in primary cultures of rat CP epithelial cells [45]. PCFT may assist in this FRα-mediated transport by exporting folates from exosomes and acidified endosomes [5, 17, 55]. The critical role of FRα in modulating cerebral folate uptake is further exemplified by disorders of CFD, which are characterized by impaired FRα function, resulting in suboptimal brain folate levels [16, 40]. Although the role of FRα and PCFT in regulating folate uptake at the CP has been established, the contribution of RFC is not well-understood. As RFC is expressed at the apical brush-border membrane of the CP epithelium, it may also assist in CNS folate delivery by facilitating folate efflux into the CSF [3, 17, 43]. As studies investigating the possible alternate pathways of folate transport in the brain are limited, the aim of this study was to further characterize the localization of the folate transport systems in the murine CNS, to provide insight on the potential contributions of the various brain cellular compartments in overall brain folate delivery.

Impairments in folate transport at the BCSFB can result in severe pediatric neurological impairments, which are characteristic of CFD and HFM [5, 12]. In children with CFD, some of the major clinical features including abnormal brain myelination, psychomotor regression, and ataxia begin to appear at 2–3 years of age [32]. HFM is characterized by defective PCFT function resulting in impaired folate absorption at the gastrointestinal tract, as well as at the BCSFB [23, 31]. Despite functional FRα and RFC expression, children with HFM present with severely low CSF folate levels (< 5 nmol/L). In HFM, symptoms appear much earlier than seen in CFD, with neurological impairments detected as early as a few months after birth [23]. This discrepancy in symptom onset may be attributed to the protective effect of the BBB, where folates can be supplied from the peripheral vasculature, since systemic folate levels are normal in CFD [52]. Several studies, including the ones from our group, have revealed robust expression of both RFC and PCFT in human and rodent in vitro models of the BBB, as well as evidence of 5-methyltetrahydrofolate transport across the brain microvessel endothelium in vivo [2–4, 6, 47]. Despite its protective role, folate transport activity at the BBB under physiological conditions may not be sufficient to prevent neurological impairments seen in CFD, as these symptoms eventually begin to manifest despite the delay in onset [52]. Our group has extensively investigated the transport of folates at the BBB, to determine whether modulating folate transport at this interface may increase overall cerebral folate uptake [2–5]. We have recently demonstrated the effects of RFC upregulation in vivo*,* in mice lacking FRα, as treatment with the VDR-activating ligand calcitriol resulted in significant increases in brain folate levels in KO mice, remarkably restoring levels to those seen in wildtype mice [2]. Interestingly, significant increases in brain folate levels were observed despite only modest increases in RFC gene expression in the isolated brain capillaries (representative of the BBB) of calcitriol-treated FRα KO mice [2]. These results suggest that an increase in folate transport at additional brain cellular compartments may have contributed to increases in overall CNS folate uptake. This is supported by recent mouse RNAseq data which detected RFC and PCFT gene expression in astrocytes, neurons and microglia, as well as leptomeningeal cells of the AB [20, 50]. Furthermore, while both the BCSFB and BBB have been established as two potential routes of blood-to-brain folate transport, it is unclear whether in addition to the exosomal transport of folates, there may be alternative mechanisms of folate transport into brain parenchymal cells [17].

At the mouse BCSFB and BBB, our present studies revealed localization of RFC, PCFT and FRα through immunohistochemical analysis, which supports previous data in the literature and from group [2, 3, 17]. Of particular interest is the localization pattern of FRα at the mouse BCSFB, which reveals robust straining on the apical side of the CP epithelium, with minor staining observed on the basolateral side. Our localization data is consistent with previous studies conducted in human and rodent tissues, which also reveal preferential localization of FRα on the apical side [17, 29, 38]. In isolated mouse CP tissue, gene and protein expression of the primary folate transport systems was confirmed, with significant FRα gene and protein expression detected. As the entire CP was dissected from the mouse brain, tissue preparations may have been contaminated with other cell types located in the CP (i.e., ependymal cells, connective tissue, etc.), possibly contributing to gene and protein expression. We also detected gene and protein expression of RFC, PCFT and FRα in primary cultures of mouse brain microvascular endothelial cells, validating our previous findings [2, 3]. It is important to note that in humans, FRα has not been reported to be expressed at the BBB, which may be due to species-specific differences in receptor localization [3]. Throughout our studies, western blot analysis revealed multiple proteins bands for both RFC (58–75 kDa) and PCFT (50–65 kDa). Multiple migratory bands that are observed may be a result of post-translational modifications of these transmembrane proteins, including differential glycosylation of N-linked glycosylation sites [3, 37, 46].

At the AB, which represents an additional blood–CSF barrier, localization of the folate transport systems was also investigated. To differentiate the arachnoid mater from the pial layer, mouse brain sections were stained with the epithelial marker e-cadherin, which is highly expressed by arachnoid epithelial cells [18]. At this site, the expression of the folate transport systems had not previously been thoroughly examined. A few studies have reported RFC gene expression in human arachnoid tissue, and RFC protein expression in rat leptomeningeal tissue [49, 51]. Consistent with the single-cell mouse RNAseq data, our studies revealed localization of RFC and PCFT at the mouse AB, with gene and protein expression also confirmed in an immortalized AB mouse cell line [20, 50]. The membrane localization of RFC and PCFT was also detected in the immortalized mouse AB cell line through immunocytochemical analysis, as verified by their similar localization to the plasma membrane marker Na^+^/K^+^-ATPase α. Due to the physiological pH typically observed throughout the CNS, RFC may likely function as the prominent folate transporter at the AB, as PCFT displays optimal activity in an acidic microenvironment [55]. In our studies, FRα localization or expression was not detected at the mouse AB. The absence of FRα in arachnoid tissue was also observed by Jimenez et al*.*, where FRα gene expression was not detected in arachnoid tissue extracts from ED18 rat fetuses [21]. This lack of FRα expression may also be attributable to age-dependent changes in FRα expression during embryonic development, which has been previously observed at the CP [40]. In recent years, the expression of drug transporters at the AB has become increasingly recognized, with expression of many notable transporters such as breast cancer resistance protein (BCRP) and peptide transporter 2 (PEPT2) displaying more abundant expression at the AB compared to the CP epithelium [41]. Relative to the total CSF volume in the CNS, only a small volume of CSF exists in the ventricles, in contrast to the large volume of CSF circulating in the subarachnoid space facing the AB; therefore, it is possible that the AB may play a major role in CSF regulation and folate transport [35, 51]. This is evidenced in the study by Zhang et al*.*, which revealed a distinct CSF-to-blood efflux pathway of organic anionic drugs at the AB (independent of the CP), by organic anion transporters (Oat) 1 and 3, highlighting the role of the AB in maintaining overall CSF homeostasis through active transport mechanisms [51]. Future studies must be conducted to examine the polarized localization of RFC at the AB, to elucidate if this transporter may indeed assist in extracting folates from the dural vasculature, to be later transported into the CSF**.** As observed in the BBB, RFC activity at the AB may not be sufficient to provide adequate folate levels when folate transport at the BCSFB is impaired, thus examining the transcriptional regulation of RFC by VDR or NRF-1 at the AB will aid in determining whether induction of RFC can enhance folate delivery into the CSF [2–4].

Beyond the BBB and BCSFB, the precise mechanisms by which folates are further transported into various brain cellular compartments have not been extensively investigated. As previously described, it is postulated that folates are further delivered into the cells of brain parenchyma through FRα-mediated exosomal transport [17]. In the study by Grapp et al*.*, mice injected with purified exosomes derived from FRα-transfected Z310 cells (or FRα-positive exosomes) showed the presence of these exosomes in neurons and astrocytes, demonstrating that the delivery of folates into brain parenchyma may not require additional carrier mediated transport [17]. Nevertheless, it has been suggested that RFC may be involved in the neuronal transport of folates, with RFC localization previously detected in mouse neurons, particularly in axons and dendrites, as well as epithelial cells of the spinal canal [43]. Additional studies by Cai and Horne demonstrated carrier-mediated transport of the active folate derivative 5-formyltetrahydrofolate in both primary cultures of rat astrocytes as well as primary cultures of rat cerebellar granule cells, indicating that RFC and PCFT may play a more significant role in brain parenchymal folate uptake [9, 10]. Evidence of FRα expression in brain parenchyma is inconclusive, as the single-cell mouse RNAseq data did not detect FRα expression in astrocytes, microglia or neurons [20, 50]. However, in a mouse hippocampal neuron cell line (HT-22), Yang et al. observed gene expression of all three folate transport systems [48]. Mann et al. also reported FRα localization in rat neurons, but this may be a result of species-specific differences [3, 26]. In our studies, immunohistochemical analysis revealed localization of RFC and PCFT, but not FRα, in astrocytes, microglia, and neurons. In primary cultures of mouse neurons and mouse mixed glial cells, gene expression of RFC and PCFT was detected, with very low levels of FRα gene expression also observed. Detection of FRα gene expression may be due to contamination of cultures with oligodendrocytes, which have been reported to express FRα [20, 50]. Western blot analysis revealed RFC and PCFT, but not FRα, protein expression in primary cultures of mouse neurons and mixed glial cells, which is consistent with our localization and gene expression data along with the single-cell mouse RNAseq data [20, 50]. These results together suggest that the low affinity transporters (RFC, PCFT) may serve as an additional mechanism of folate transport into neural cells in parallel to exosomal transport. Similarly seen with the BBB and AB, PCFT may be a less relevant folate transporter in brain parenchyma, due to the low pH required for optimal activity. To further delineate the role of PCFT in brain parenchymal folate transport, its subcellular localization must be examined, as PCFT may be localized in intracellular compartments within brain parenchymal cells. PCFT may contribute to folate uptake in these cells by assisting in folate export from FRα-containing exosomes that originate from the CP epithelium [17].

## Conclusions

Taken together, our studies have demonstrated novel localization of the low affinity folate transporters RFC and PCFT at the AB and in cells of brain parenchyma (i.e., in astrocytes, microglia, neurons) potentially serving as additional routes of folate delivery in the CNS. These studies provide insight into the mechanisms by which folates may be transported and localized within brain parenchyma independent of FRα-mediated transport. As demonstrated at the BBB, augmenting functional expression of RFC and PCFT through activation of transcription factors (i.e., VDR, NRF-1) at these cellular compartments may result in increased overall folate uptake throughout the CNS when FRα transport at the BCSFB is compromised. These studies may be critical in further understanding the role of folate transport in the context of neurodevelopment, as well as in drug discovery for the treatment of disorders associated with brain folate deficiency.

## Supplementary Information

**Additional file 1.** Cellular localization of vimentin in immortalized cell cultures of mouse AB. Cells were stained with the following: DAPI nuclear marker, or anti-vimentin (1:50) (Panel 1) No primary antibody was used as a negative control (Panel 2). Sections were visualized using confocal microscopy (LSM 700; Carl Zeiss) operated with ZEN software using an oil-immersion 63x lens.

## Data Availability

The datasets used and/or analysed during the current study are available from the corresponding author on reasonable request.

## Abbreviations

AB: Arachnoid barrier
ABC: ATP-binding cassette
Aqp-1: Aquaporin-1
ASD: Autism spectrum disorder
BBB: Blood–brain barrier
BCRP: Breast cancer resistance protein
BCSFB: Blood–cerebrospinal fluid barrier
BSA: Bovine serum albumin
CFD: Cerebral folate deficiency
CNS: Central nervous system
CP: Choroid plexus
DMEM: Dulbecco’s modified Eagle’s medium
ED: Embryonic day
FBS: Fetal bovine serum
FRα: Folate receptor alpha
FRΑA: Folate receptor autoantibodies
HFM: Hereditary folate malabsorption
KSS: Kearns Sayre syndrome
NRF-1: Nuclear respiratory factor 1
OAT: Organic anion transporter
OCT: Optimal cutting temperature
PBS: Phosphate buffer saline
PCFT: Proton-coupled folate transporter
PFA: Paraformaldehyde
PDL: Poly-d-lysine
P-gp: P-glycoprotein
PEPT: Peptide transporter
PQQ: Pyrroloquinoline quinone
qPCR: Real-time quantitative PCR
RFC: Reduced folate carrier
SDS: Sodium dodecyl sulfate
SLC: Solute carrier
VDR: Vitamin D receptor
