# Supplementary material for: Novel localization of folate transport systems in the murine central nervous system
Source: Fluids Barriers CNS. 2022 Nov 23;19:92. doi: 10.1186/s12987-022-00391-3 (PMC9686069; doi:10.1186/s12987-022-00391-3)
Supplement: Supplementary file 1 — Additional file 1. Cellular localization of vimentin in immortalized cell cultures of mouse AB. Cells were stained with the following: DAPI nuclear marker, or anti-vimentin (1:50) (Panel 1) No primary antibody was used as a negative control (Panel 2). Sections were visualized using confocal microscopy (LSM 700; Carl Zeiss) operated with ZEN software using an oil-immersion 63x lens. [file 12987_2022_391_MOESM1_ESM.docx]

**Supplemental Data:**

**
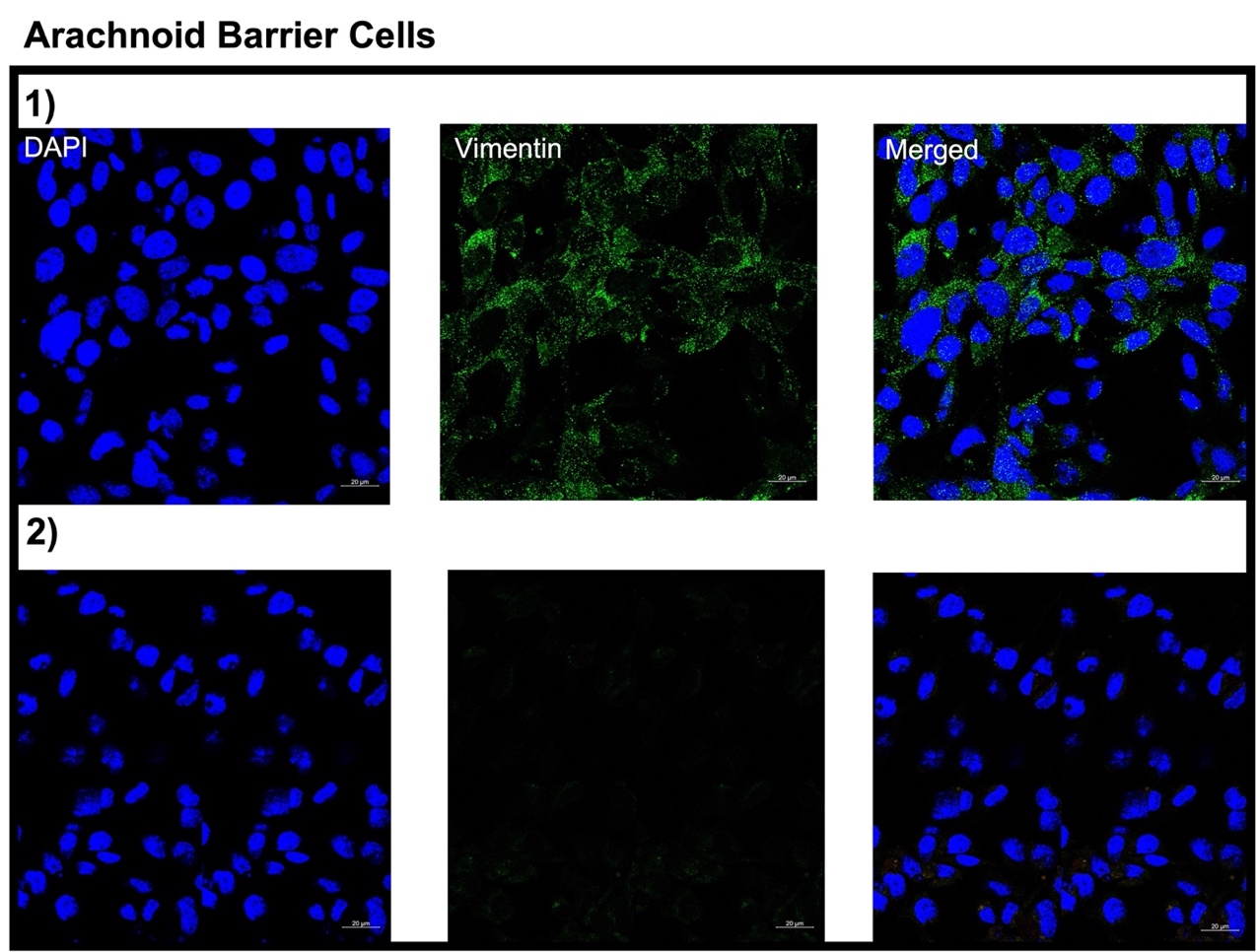
**

**Supplementary Figure 1:** Cellular localization of vimentin in immortalized cell cultures of mouse AB. Cells were stained with the following: DAPI nuclear marker, or anti-vimentin (1:50) (Panel 1) No primary antibody was used as a negative control (Panel 2). Sections were visualized using confocal microscopy (LSM 700; Carl Zeiss) operated with ZEN software using an oil-immersion 63x lens.
